# Intra-Individual Response Variability Assessed by Ex-Gaussian Analysis may be a New Endophenotype for Attention-Deficit/Hyperactivity Disorder

**DOI:** 10.3389/fpsyt.2014.00197

**Published:** 2015-01-12

**Authors:** Marcela Patricia Henríquez-Henríquez, Pablo Billeke, Hugo Henríquez, Francisco Javier Zamorano, Francisco Rothhammer, Francisco Aboitiz

**Affiliations:** ^1^Department of Clinical Laboratories, Pontificia Universidad Católica de Chile, Santiago, Chile; ^2^Cognitive Neurosciences Laboratory, Department of Psychiatry, Pontificia Universidad Católica de Chile, Santiago, Chile; ^3^Centro de Investigación en Complejidad Social (CICS), Facultad de Gobierno, Universidad del Desarrollo, Santiago, Chile; ^4^Medical Technology School, Universidad Mayor, Santiago, Chile; ^5^Instituto de Alta Investigación, Universidad de Tarapacá, Arica, Chile

**Keywords:** ex-Gaussian analysis, ADHD, intra-individual variability, endophenotypes, response time

## Abstract

Intra-individual variability of response times (RTisv) is considered as potential endophenotype for attentional deficit/hyperactivity disorder (ADHD). Traditional methods for estimating RTisv lose information regarding response times (RTs) distribution along the task, with eventual effects on statistical power. Ex-Gaussian analysis captures the dynamic nature of RTisv, estimating normal and exponential components for RT distribution, with specific phenomenological correlates. Here, we applied ex-Gaussian analysis to explore whether intra-individual variability of RTs agrees with criteria proposed by Gottesman and Gould for endophenotypes. Specifically, we evaluated if normal and/or exponential components of RTs may (a) present the stair-like distribution expected for endophenotypes (ADHD > siblings > typically developing children (TD) without familiar history of ADHD) and (b) represent a phenotypic correlate for previously described genetic risk variants. This is a pilot study including 55 subjects (20 ADHD-discordant sibling-pairs and 15 TD children), all aged between 8 and 13 years. Participants resolved a visual Go/Nogo with 10% Nogo probability. Ex-Gaussian distributions were fitted to individual RT data and compared among the three samples. In order to test whether intra-individual variability may represent a correlate for previously described genetic risk variants, VNTRs at *DRD4* and *SLC6A3* were identified in all sibling-pairs following standard protocols. Groups were compared adjusting independent general linear models for the exponential and normal components from the ex-Gaussian analysis. Identified trends were confirmed by the non-parametric Jonckheere–Terpstra test. Stair-like distributions were observed for μ (*p* = 0.036) and σ (*p* = 0.009). An additional “DRD4-genotype” × “clinical status” interaction was present for τ (*p* = 0.014) reflecting a possible severity factor. Thus, normal and exponential RTisv components are suitable as ADHD endophenotypes.

## Introduction

Symptom-based diagnostic systems such as the current versions of the Diagnostic and Statistical Manual of Mental Disorders (DSM) (1) and the International Classification of Diseases (ICD) (2) have proved to be useful in the clinical approach to the neuropsychiatric patient and in epidemiological settings. Notwithstanding, in opinion of some authors, their massive utilization in molecular-genetic studies has probably hampered (or even obstructed) the identification of susceptibility genes for neuropsychiatric disorders, due to the inherent multidimensionality of phenotypes defined by them (3). This multidimensionality probably hides genetic heterogeneity, non-genetic phenocopies, and complex networks of gene–gene and gene–environment interactions, among other confounding phenomena, lowering the statistical power of association studies (3–5).

Taking this into account, it has been proposed to replace symptom-based phenotypes by quantifiable markers of liability or “risk” for a specific disorder (4). These markers, generically called “endophenotypes” constitute a more “direct” expression of the gene effect, since they conceptually lie between the gene and the disorder and – in consequence – are influenced by fewer genetic and environmental variables than the disorder itself. In addition to their potential role improving the statistical power of molecular-genetic studies, endophenotypes should be a valuable tool when studying how the already-known genetic risk variants are related to the neurobiological and neuro-physiological phenotypes that underlie psychiatric disorders, which could be the first step to elucidate the specific domains of brain function influenced by these variants. This kind of approach has been successfully applied, for example, in the functional characterization of risk genetic variants for psychotic and affective disorders (6–8).

In the last few years, researchers have started to explore the potential of some neuro-cognitive and electrophysiological/radiological markers as endophenotypes for attentional deficit/hyperactivity disorder (ADHD). Among them, intra-individual variability [defined as short-term changes in behavior that are signed as moment-to-moment fluctuations in task performance (9, 10)] seems to be especially promising. Elevated intra-individual variability of the response times (RTisv) in ADHD patients is one of the most consistent findings across the ADHD literature and has been documented in task assessing working memory (11), attention (12, 13), inhibitory control (12, 14), and choice discrimination (15). Interestingly, the magnitude of the between-group differences reported tends to be larger than most other neuropsychological parameters studied to the date (16). Additionally, intra-individual variability is in close agreement with some of the criteria proposed by Gottesman and Gould to facilitate the exclusion of spurious endophenotypes and to increase the likelihood of identifying stronger associations with genetic factors (4, 17). In this context, it is worth to mention two important lines of evidence: (1) studies showing that affected relatives of ADHD children present greater intra-individual variability compared to controls (18, 19) and (2) studies supporting the heritability of intra-individual variability (15, 18, 20).

Traditionally, intra-individual variability has been estimated by collapsing responses across the entire time interval of the task, resulting in a single point parameter such as the standard deviation of the response time (RTSD) or the coefficient of variation of the response time (RTCV), which should capture and represent how data spread around the mean value. This method implies to lose a significant amount of specific information regarding the distribution of response times (RTs) along the task, with eventual effects on statistical power when comparing RTisv among clinical groups. Particularly, this method does not recognize the asymmetrical positive skew that occasional lapses in attention are expected to produce in the RTs distributions (21).

During the last decade, the field has moved toward new statistical approaches aiming to obtain more complete and specific characterizations of the RTisv. Among these approaches, the ex-Gaussian analysis on the RTs has provided enlightening results regarding the dynamic nature of intra-individual variability in ADHD. Basically, the ex-Gaussian analysis is based on the convolution of an exponential and a Gaussian function, obtaining three parameters: μ (mu), corresponding to the mean of the normal component, σ (sigma), corresponding to the SD of the normal component, and τ (tau), which describes the mean of the exponential component. When fitting ex-Gaussian distributions to RT data, greater values for τ indicate a higher frequency of excessively long RTs (22). In general, most of the studies that have applied ex-Gaussian analysis on RT data from ADHD patients showed elevation of τ component in the ADHD group (11, 22–27). This finding is consistent with lapses in attention, due to a defective effort control mechanisms (22).

In this work, we hypothesize that RTisv – expressed as the τ and σ components of the ex-Gaussian distribution of RTs – may be a potential endophenotype for ADHD. In order to address this hypothesis, we first explored whether healthy siblings of ADHD patients may present intermediate values of intra-individual variability in front of a motor inhibition task (Go/NoGO) when compared to ADHD patients and typically developing children (TD) without family history of ADHD, and so that, whether intra-individual variability may follow the hypothetical distribution postulated for endophenotypes. As a second step, we explored whether RTisv may represent a phenotypic correlate for previously described genetic risk variants for ADHD in the genes encoding for the dopaminergic receptor D4 (*DRD4*) and for the dopaminergic transporter 1 (*SLC6A3/DAT1*). We choose the aforementioned genetic risk variants based on previous evidence suggesting that RTisv may be associated to dopamine dysfunction (28–30).

To the best of our knowledge, this is the first study applying the ex-Gaussian approach to simultaneously characterize the performance of ADHD patients, their asymptomatic first degree relatives, and TD without family history of ADHD. Additionally, after an exhaustive search, we did not find other studies applying ex-Gaussian analysis in order to evaluate intra-individual variability as a phenotypic correlate to genetic variants in *DRD4* and *SLC6A3/DAT1* genes, which has been previously linked to ADHD.

## Materials and Methods

### Participants

In order to evaluate eventual differences in regards to the RT distribution observed among ADHD patients, their non-affected siblings, and TD without family history of ADHD, we evaluated a total of 55 subjects, corresponding to 20 discordant sibling-pairs and 15 unaffected children from the general population. Sibling-pairs were originally recruited as part of a parallel genetic association study started in 2003 (31). This study included families from the Great Santiago Area, referred from general psychiatric and neurological outpatient services directed to medium-income population. Unaffected children without family history of ADHD were recruited from a medium-income school from the same socio-economical and geographical area. Cases and controls belong to a narrow age-range (ranged between 8 and 13 years) in order to avoid age-confounding effects on performance/neurobehavioral measurements. Groups were comparable by age distribution (ADHD group: mean = 11.2 years, SD = 2.47; sibs group: mean = 11 years, SD = 2.35; unaffected children from general population: mean = 11.6 years, SD = 0.9 *F* = 0.25; *p* = 0.77) and gender distribution [ADHD group: 17 boys and 3 girls; sibs group: 13 boys and 7 girls; unaffected children from general population: 9 boys and 6 girls χ^2^(2) = 3.1; *p* = 0.21].

Inclusion criteria for ADHD children were as follows: ADHD combined subtype according to DSM (32), age between 8 and 13 years, having at least one unaffected sibling in the same range of age and good response to stimulant medication. We included the latest criteria in order to reduce clinical heterogeneity in our size-restricted sample. Operationally, we defined “good response to medication” as a clinically significant improvement in symptomatology reported by parents in an interview with a competent specialist and documented as at least 20% reduction in the Conners’ Abbreviated Parent–Teacher Questionnaire for ADHD symptoms (33). All patients included in this study were treated with d-amphetamine or methylphenidate, both in doses ranging 10–30 mg/day. A wash-out period of 24 h free of medication was required before the neuropsychological assessment.

In the case of the groups composed by non-affected siblings and TD from the general population, inclusion criteria were age between 8 and 13 years old and absence of ADHD or any other psychiatric morbidity according to DSM-IV criteria. Additionally, a negative familiar history of ADHD was required in the TD group. Familiar history of ADHD was explored in first degree relatives (siblings and parents) of TD participants by means of a semi-structured interview assessing presence/absence of DSM-IV criteria during childhood. Children with neurological deficit at physical examination and/or abnormal baseline electroencephalography (EEG) were excluded from the study. All aforementioned inclusion/exclusion criteria were assessed according to current ADHD diagnosis guidelines by a competent specialist (child psychologist or child neurologist).

We only included ADHD patients and their unaffected siblings for genotyping analysis. This design was chosen in order to control for an eventual population stratification effect. As additional advantage, discordant sibling-pairs share most of the psychosocial and familial factors that might interact with a potential genetic predisposition. Thus, for the genetic part of the study, 40 participants (corresponding to 20 siblings-pairs) were assigned to “Risk” or “Non-Risk” genotype groups accordingly with the criteria described in Section “Group Comparisons.”

### Genotyping

Genomic DNA was isolated from peripheral blood lymphocytes by standard methods and amplified by polymerase chain reaction (PCR) to identify the VNTR of the *DRD4* and *SLC6A3* loci according to the protocols described by Lichter et al. (34) and by Cook et al. (35), respectively. PCR products were visualized by electrophoresis in 3% agarose gels. Details on the PCR primers sequences have been previously reported by our group (36).

### Task and procedures

Details on the Go–NoGo task performed in this study have been previously reported by our group (36). Briefly, all participants responded to an 8 min long Go–NoGo Task with a Go:NoGo rate of 9:1. Stimuli corresponded to green (Go) and red (NoGo) circles of 2 cm in diameter presented at the center of a black screen during 300 ms, with an inter-trial period of 1,000 ms. A pseudorandom process ensured that at least six Go stimuli were presented before each NoGo stimulus. The total task comprised the presentation of 360 Go and 40 NoGo stimuli. The instruction was to click in a console as fast as possible, with the dominant hand, after every Go stimulus, but to inhibit the response in front of NoGo stimuli. A treatment wash-out period of 24 h was required in all ADHD patients previous to task performance. No additional tasks or procedures were performed in the same session. No exclusions due to task non-compliance were registered.

### Data analysis

#### Data treatment for ex-Gaussian analysis

Data corresponding to the response time (RT) in front of all Go stimuli were obtained from each subject. Only correct responses were computed in order to determine the parameters of the ex-Gaussian distribution of RT, according to the data-analysis protocol originally described by Leth-Stevenson et al. (22). Additionally, any single RT of <100 ms was considered as accidental key press and was excluded. Ex-Gaussian distributions were fitted to RT data using the *R* statistical package (37), and μ, σ, and τ parameters were computed for each participant. The number of RT observations used for each ex-Gaussian fit depended on the accuracy of responding (mean = 287 trials, range = 97–357). Finally, in order to assess the Goodness-of-fit of the ex-Gaussian models, the empirical distribution for each child was compared to a random ex-Gaussian distribution using the values for μ, σ, and τ adjusted for the same subject. Comparison was performed by means of the Kolmogorov–Smirnov Test. There were no significant differences between empirical and ex-Gaussian distributions for any one of the analyzed subjects.

#### Group comparisons

In order to compare the characteristics of ex-Gaussian distributions among ADHD children, their non-affected sibs, and TD from the general population (TD), we adjusted independent general linear models for μ, σ, and τ, using age and sex as co-variates and “clinical group” (ADHD, non-affected sib, or TD) as an ordinal variable, with ADHD as the highest ordinal value and TD as the lowest. Additionally, we confirmed the statistical significance of the detected trends by means of the non-parametric Jonckheere–Terpstra test to evaluate trend in the data (e.g., ADHA > non-affected sibs > TD). Significance was defined as alpha = 0.05. For multiple comparisons, *p*-values after *post hoc* analysis are reported.

When comparing the characteristics of the ex-Gaussian distributions regarding to the presence/absence of genetic risk variants previously linked to ADHD in the *DRD4* and *SCL6A3* genes, on the other hand, we applied three consecutive approaches. First, eventual differences in μ, σ, and τ between the genetic risk and non-risk groups were preliminary explored by means of non-parametrical methods (Wilcoxon Rank Test). In a second step, significance levels were adjusted by age, sex, and clinical status by means of general linear models. Finally, the effect of an interaction between the “clinical status (presence/absence of ADHD) and genotype group (risk/control) was explored by general linear models for μ, σ, and τ, with the independent variables “genetic risk group” (risk versus control group), age, sex, clinical status, and “genetic risk group × clinical status” interaction. As mentioned above, in order to control for population stratification, only sib-pairs were included for genetic analysis.

“Genetic risk groups” were defined as follows: (a) For DRD4; the “control” or “non-risk” group was comprised by homozygous for the DRD4 4-repeat allele. A total of 22 subjects were assigned to this group and 9 of them were ADHD patients. The “risk” group, on the other hand, was constituted by carriers of at least one copy of alleles 7R (*n* = 11; 3 of them in homozygous state) or 2R (*n* = 7; two of them in homozygous state). This group included 18 subjects and 11 subjects presented ADHD among them. There was no association between allele possession for Exon 3 VNTR of *DRD4* and clinical status [χ^2^ (1) = 0.9, *p* = 0.34]. We chose to include carriers of the 7R and 2R alleles in the “risk group” based on previous reports of association between ADHD and these variants in different populations (38–40). Additionally, some *in vitro* studies have suggested that receptors encoded by 2R and 7R alleles mediate a reduced response to dopamine in comparison with the 4R allele (41). (b) For *SLC6A3*; the “risk group” were comprised by homozygous for 10R (*n* = 24; 13 of them were ADHD patients) and the “control” or “non-risk” was constituted by subjects with any other genotype (*n* = 15; 6of them were ADHD patients). The latter group comprised 14 participants with 9R/10R genotype and 1 homozygous for the 9R allele. There was no association between allele possession for 3′ UTR VNTR of *SLC6A3* and clinical diagnosis of ADHD, χ^2^(1) = 0.28, *p* = 0.59.

### Ethical issues

All procedures performed as part of this study were approved by the Ethics Committee of the Pontificia Universidad Catolica de Chile. The study was fully explained to children and their parents, and they both agreed to participate by signing written consent forms.

## Results

### Comparison of ex-Gaussian distributions among ADHD children, their non-affected sibs, and TD from the general population

**Figure 1:**
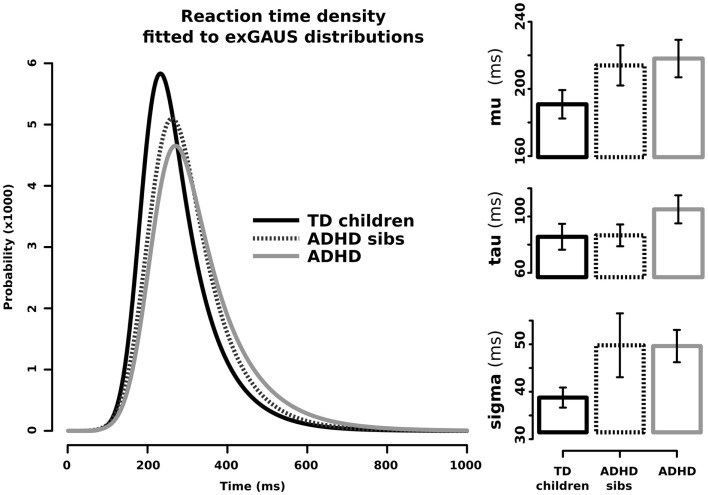
**Ex-Gaussian distributions for response times in ADHD children, their unaffected sibs, and typically developing children without family history of ADHD**. Left side: RT density fitted to ex-Gaussian distributions. Right side: μ (mu), σ (sigma), and τ (tau) components in the three studied groups; bars represent SE for the media.

Figure 1 shows the graphical representation and values for μ, σ, and τ when adjusting ex-Gaussian distributions to the RT data from ADHD patients (solid gray line), non-affected sibs (dotted line), and TD without family history of ADHD (solid black line). In order to statistically explore eventual differences among these groups, we performed linear models for the aforementioned parameters, with clinical group as the ordinal variable. Results from this analysis are summarized in Table 1. There was a statistically significant effect of the independent variable “clinical group” on the parameters μ (corresponding to the mean of the Gaussian/normal component of the distribution; *p*-value = 0.015). Since models were adjusted with ADHD as the highest ordinal value and TD as the lowest, we can assume that μ presented with the stair-like distribution typically expected for endophenotypes. A similar interesting trend was observed for σ (corresponding to the SD of the Gaussian/normal component), although it did not reach statistical significance after controlling for age and sex (*p*-value = 0.052). We did not find a statistically significant effect for “clinical group in the models adjusted for τ. Similar results were obtained using Jonckheere–Terpstra test to evaluate the trend ADHD > non-affected sibs > TD (σ: JP = 587, *p*-value = 0.009, predictive strength *r* = 0.35; μ: JP = 559, *p*-value = 0.0358, predictive strength *r* = 0.36; τ: JP = 523, *p*-value = 0.13).

**Table 1:** **Summary of estimated effects in general linear models adjusted for mu, sigma, and tau parameters**.

Model for…	Independent variable	Estimated effect	SE	*t*	Effect-size *r*	*p*-value
μ	Clinical group	19.09	7.61	2.5	0.33	0.015
	Age	−6.96	2.88	−2.41	0.31	0.019
	Sex	−28.82	13.28	−2.16	0.28	0.035
σ	Clinical group	6.33	3.19	1.99	0.26	0.052
	Age	−3.16	1.21	−2.61	0.34	0.011
	sex	−9.32	5.56	−1.65	–	0.1
τ	Clinical group	3.67	7.74	0.52	–	0.6
	Age	−7.45	2.93	−2.5	0.33	0.015
	sex	−2.8	13.5	−0.21	–	0.83

### Comparison of ex-Gaussian distributions regarding to the presence/absence of genetic variants previously linked to ADHD in the *DRD4* and *SCL6A3* genes

**Figure 2:**
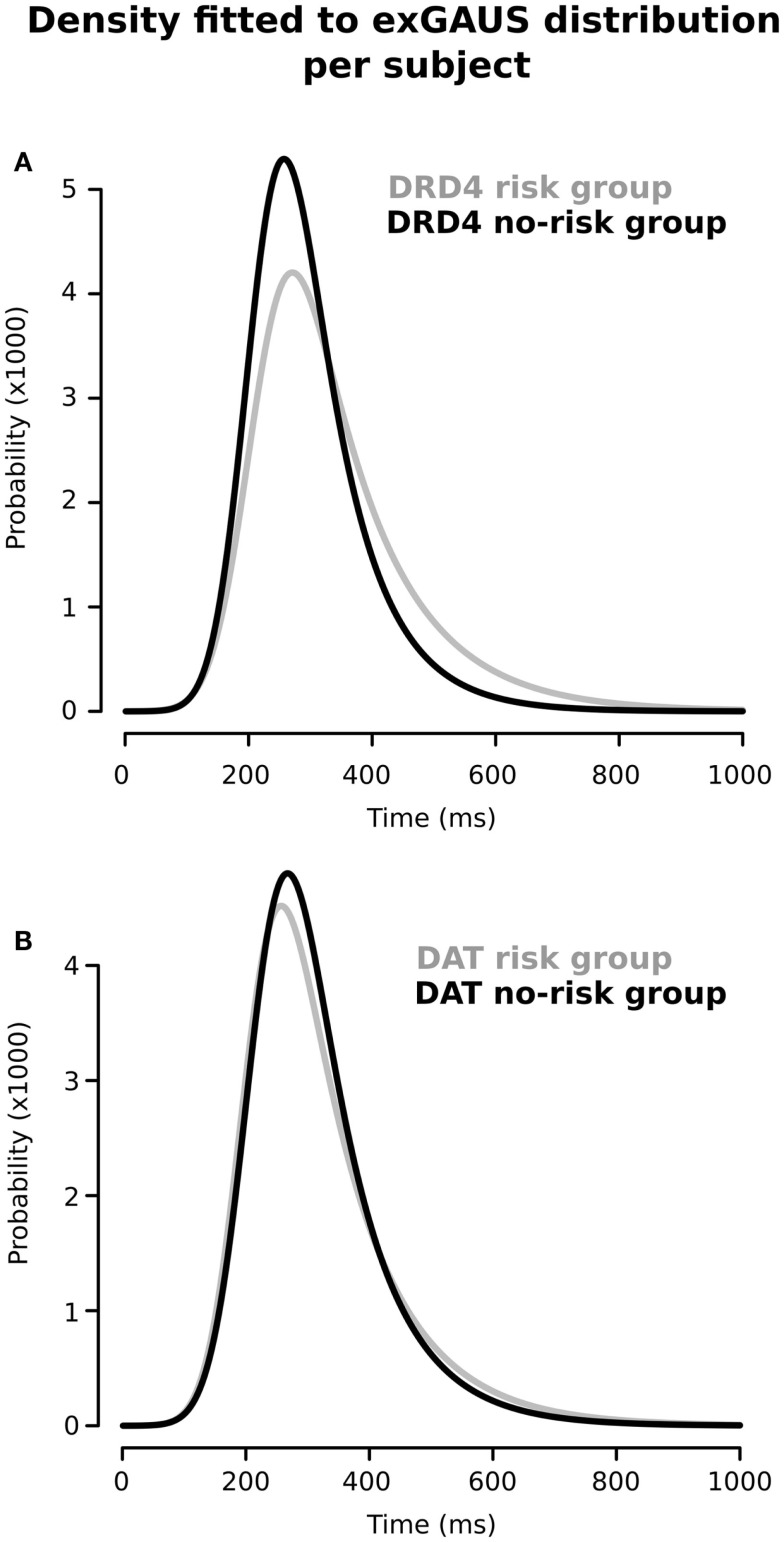
**Ex-Gaussian distributions for response times according to the presence/absence of previously described genetic risk variants for (A) *DRD4* and (B) *SCL6A3*/*DAT1***. Black line: absence of risk alleles; gray line: presence of at least one risk allele (for a detailed description on group assignment criteria, please refer to Sections “Materials and Methods,” and Sections “Group Comparisons”).

**Figure 3:**
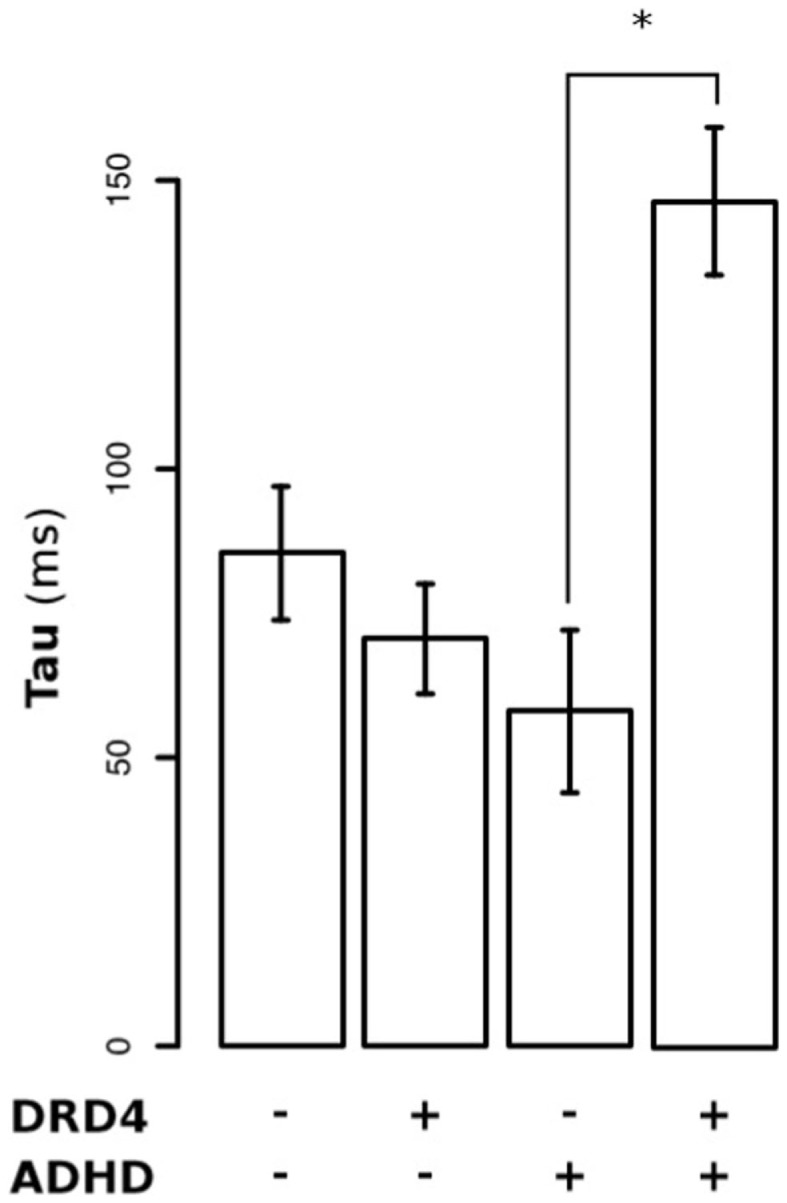
**Differential effect of DRD4 genotype on τ (tau) among ADHD children versus their asymptomatic siblings**.

Figure 2 shows the RT distributions obtained when we classified and analyzed the subjects in regards to their “genotype group” for *DRD4* (2A) and *SCL6A*3 (2B). In the case of *DRD4*, subjects were classified depending on the presence/absence of VNTR variants previously linked to ADHD in the exon III of *DRD4*, with carriers of at least one copy of the 7R or 2R allele considered as the risk group and homozygous for 4R as the controls/non-risk group. The ex-Gaussian distribution adjusted among subjects from the genetic risk group is more skewed than the ex-Gaussian distribution for *DRD4-4R* homozygous, which is expressed as a bigger value for τ (unadjusted *p* = 0.013; see Table 2 for a summary of comparisons between *DRD4/SCL6A3* genotype groups). Interestingly, linear models demonstrated a statistically significant effect of the “genotype group” × “clinical status” interaction exclusively on τ (*p*-value = 0.014; see Table 3 for a summary of interaction models). This interaction reflects a greater τ among ADHD subjects carrying *DRD4*-risk alleles in comparison to *DRD4*-4R homozygous (Kruskal–Wallis rank sum test, χ^2^ = 0.9562, df = 1, *p*-value = 0.01467, effect-size *r* = 0.44, and *post hoc p*-value: 0.03196; see Figure 3). In the case of *SCL6A3*, on the other hand, we did not observe any significant effect of genotype on any of the ex-Gaussian parameters analyzed (see Figure 2; Table 2).

**Table 2:** **Ex-Gaussian parameters in subjects presenting “risk” versus subjects presenting “non-risk” alleles for *DRD4* and *SCL6A3/DAT1* genes**.

Gene	Parameter (ms)	Risk group	Non-risk group	Unadjusted *p*-value^a^	Adjusted *p*-value^b^
*DRD4*	μ	212.2	209.1	0.96	0.56
	σ	52.3	47.3	0.24	0.94
	τ	122.7	83.2	0.013*	0.14
*SCL6A3/DAT1*	μ	214.8	201.8	0.48	
	σ	51.2	47.2	0.92	
	τ	97.3	117.2	0.26	

*Indicates a significance level of 0.05 or less.

*^a^Unadjusted *p*-value was obtained by means of Wilcoxon sum rank test*.

*^b^Presented *p*-values are adjusted by age, sex, and clinical status (ADHD, healthy sibs, and/or TD children)*.

**Table 3:** **Estimated effects for all co-variates included in the linear regression models adjusted for μ, σ, and τ**.

Model for…	Independent variable	Estimated effect	SE	*p*-value	Effect-size, *r*
μ	Genotype	−23.75	21.98	0.29	
	Clinical status	11.52	22.45	0.61	
	Age	−5.73	3.81	0.17	
	Sex	−19.73	20.23	0.34	
	Genotype × clinical status	24.97	28.76	0.39	
σ	Genotype	−6.17	10.61	0.56	
	Clinical status	1.5	10.83	0.89	
	Age	−3.35	1.84	0.07	
	Sex	−9.04	9.76	0.36	
	Genotype × clinical status	11.66	13.87	0.4	
τ	Genotype	−11.87	16.01	0.47	
	Clinical status	4.78	16.36	0.77	
	Age	−5.79	2.78	0.05*	0.34
	Sex	−26.21	14.74	0.09	
	Genotype × clinical status	54.71	20.96	0.015*	0.41

*Indicates a significance level of 0.05 or less.

## Discussion

In the present study, we hypothesized that RTisv, assessed by means of the ex-Gaussian approach, is suitable as an endophenotype for ADHD. The principal aim for the first part of the study was to explore whether any of the parameters characterizing the ex-Gaussian curves fitted for ADHD patients, their healthy siblings, and/or TD children without family history of ADHD, present the “stair-like distribution” expected for an ADHD endophenotype. In the second part, we intended to explore if the parameters characterizing the ex-Gaussian distribution of RTs may correspond to phenotypic correlates for genetic variants in DRD4 and *SCL6A3/DAT1*, which have been previously linked to ADHD.

From a phenomenological perspective, ex-Gaussian analysis is able to distinguish whether differences in group performances obey to (a) a generalized slowing down of the responses times (reflected at the μ component), (b) an increased variability throughout the complete distribution (reflected at the σ component), or (c) an increased number of abnormally slow responses (reflected at the τ component). The aforementioned distinction could be mechanistically relevant, since it has been suggested that larger τ values may reflect inconsistent effort and fluctuations in attention (attentional lapses), while larger σ components may reflect deficits in motor timing and/or impaired response preparation (22, 25). Notwithstanding the potential enclosed in the aforementioned fine-grained analysis, to date, only few studies have applied this approach to explore the characteristics of responses of ADHD children. The majority of these studies have found that τ values (representing the positive skew of the RT distribution) present the highest association with ADHD in tasks requiring limited executive control, while σ became importantly correlated to ADHD when analyzing data from tasks requiring high executive/inhibition control (11, 22–24, 26, 27). Interestingly, the characteristics of the RT ex-Gaussian distribution among ADHD patients seems to be dependent on the task executive control requirements, by one hand, and on the task duration, by the other hand, since some studies applying shorter tasks (3–7 min) have failed to find higher τ component on the RT data of ADHD patients. In the same line, the duration of the inter-stimulus interval can change the cognitive process measure in the Go/No-Go task (42). A recent meta-analysis shows that long inter-stimuli intervals are more sensitive to induce low inattentive responses (43). Thus, these findings suggest that both, the rate of stimuli presentation and the duration of the task, influence the amount of low RTs generated by attentional lapses.

The Go–NoGo task used in this study demands high levels of executive/inhibition control due to extended task duration (about 8 min), low probability for the NoGo stimuli (10%), and shot inter-stimuli interval (1000 ms). In these conditions, the μ and σ components obtained after fitting ex-Gaussian curves on the RT data from ADHD patients, healthy siblings, and TD children without family history of ADHD, showed the stair-like distribution expected for endophenotypes. This finding suggests that not only ADHD children but also ADHD healthy relatives (or at least a subpopulation of them) may present alterations in response preparation and motor timing. By contrast, our experimental conditions are not particularly conducive to the appearance of attentional lapses. Hence, it was expected that we failed to find the aforementioned distribution in the case of τ component, due to our short inter-trial periods. Nonetheless, the second part of our analysis showed a statistically significant effect of the “genotype” × “clinical status” interaction for DRD4 on τ (*p*-value = 0.014). This result suggests that – in the context of a relatively long and high demanding inhibition task – attentional lapses may be predominately expressed on those ADHD patients carrying at least one copy of the 7R or 2R VNTR variants of *DRD4*. This finding is in accordance with other works, which associate DRD4 with severity of ADHD symptoms of impulsivity (44) and inattention (45). Thus, the presence of the risk alleles of *DRD4* could reflect an eventual severity mark within the ADHD group that requires further study.

Although promising, our results require further analysis and replication. Our small sample size importantly limits the statistical power of the present study. Our inclusion criteria limit the generalization of our results only to patients presenting good response to stimulant medication.

## Conclusion

Together, our results suggest that intra-individual variability is suitable as an endophenotype for ADHD. Interestingly, under our conditions of high requirements of executive/inhibitory control, only the σ component of variability presented the theoretical “stair-like distribution” for endophenotypes, while only τ (tau) seemed to be affected by the DRD4-genotype. Overall, our results emphasize the advantages of introducing new statistical methods oriented to reach a dynamic and fine-grain characterization of performance measures obtained from well-validated tasks in the analysis of new candidate-endophenotypes in psychiatric disorders. New studies are needed in order to confirm our results under different attention and inhibitory loads.

## Author Contributions

Study/experiments design: Marcela Patricia Henríquez-Henríquez, Francisco Aboitiz, and Francisco Rothhammer. Experiment execution: Marcela Patricia Henríquez-Henríquez, and Francisco Javier Zamorano. Genotyping: Hugo Henríquez and Francisco Rothhammer. Data analysis/statistical analysis: Marcela Patricia Henríquez-Henríquez and Pablo Billeke. Interpretation of results and discussion: Marcela Patricia Henríquez-Henríquez, Pablo Billeke, Francisco Aboitiz, and Francisco Rothhammer. Contributed reagents/materials/analysis tools: Marcela Patricia Henríquez-Henríquez, Pablo Billeke, Hugo Henríquez, and Francisco Rothhammer. Elaboration of the manuscript: Marcela Patricia Henríquez-Henríquez, Pablo Billeke, and Francisco Aboitiz. Critic lecture of the manuscript: Marcela Patricia Henríquez-Henríquez, Pablo Billeke, Francisco Javier Zamorano, Francisco Rothhammer, and Francisco Aboitiz.

## Conflict of Interest Statement

The authors declare that the research was conducted in the absence of any commercial or financial relationships that could be construed as a potential conflict of interest.
